# PARP inhibitors in melanoma treatment: potential, challenges, and future directions

**DOI:** 10.3389/fonc.2025.1552386

**Published:** 2025-08-06

**Authors:** Angela Anaeme, Karen Moussa, Abdallah N. Mansour, George Nassief, George Ansstas

**Affiliations:** ^1^ Division of Medical Oncology, Department of Medicine, Washington University School of Medicine in St. Louis, St. Louis, MO, United States; ^2^ School of Medicine, University of Missouri, Kansas City, MO, United States; ^3^ Athens International Master Program in Neuroscience, National and Kapodistrian University of Athens, Athens, Greece

**Keywords:** PARP inhibitors, homologous recombination deficiency, melanoma, cutaneous melanoma, synthetic lethality

## Abstract

Genome integrity is a critical driver of cellular stability, and defects in the processes that maintain genome health are potent sources of cancer progression. Homologous recombination deficiency (HRD), which damages cells through absent or erroneous repair of double-stranded DNA breaks, is a prime example of such cellular dysfunction. Poly(ADP-ribose) polymerase (PARP) inhibitors exploit these aberrancies in the cellular repair process by arresting the ability of the PARP enzyme to repair cellular and genetic damage, inducing the accumulation of DNA damage and cancer cell death. While the utility of PARP inhibitors has been established in many HRD-associated tumors — particularly breast, pancreatic, ovarian, and prostate cancer — less robust evidence exists for the efficacy of PARP inhibitors in melanoma. Increasing efforts are underway to investigate PARP inhibitors as a viable treatment option for advanced and metastatic melanoma, both as monotherapy and in combination with other agents such as immune checkpoint inhibitors and BRAF/MEK inhibitors. Though several gaps in our knowledge of the use of PARP inhibitors in melanoma still exist, promising headway is being made in our understanding of its efficacy and safety. Here, we present a review of the utility of PARP inhibitors in melanoma, current clinical trials, and future avenues for further exploration.

## 1 Introduction

One of the primary drivers of cancer development and progression is genomic instability, as well as the malfunction of pathways that maintain cellular integrity. Two critical processes that are responsible for targeting and rectifying cellular injury include homologous recombination repair (HRR) and DNA damage repair (DDR) (1, 2). HRR refers to the process by which double-stranded DNA breaks are rejoined through inter-strand crosslinks, whereas DDR encompasses the broad spectrum of cellular mechanisms by which damaged DNA is detected and repaired (2, 3). The malfunction or absence of these processes can lead to the failure of an array of processes and, ultimately, the development of cancer. Two consequences of HRR and DDR malfunction are homologous recombination deficiency (HRD), which refers to the defective and error-prone reparation of double-stranded DNA breaks, and loss of heterozygosity (LOH), which is the loss of genetic diversity at a locus due to inactivation of one allele. HRD may predispose cells to the development of LOH which, in turn, can potentiate the development of cancer if the locus involved in LOH encodes tumor suppressor genes or oncogenes (4). HRD and LOH are particularly potent drivers of the development of cancers characterized by defective DDR pathways — namely breast, pancreatic, ovarian, and prostate cancers (5–9). Thus, it is evident that the accumulation of cellular injuries that result from HRD and other repair malfunctions is central to cancer development and evolution.

Recently, new cancer therapies have emerged that exploit these deficiencies in cellular repair pathways to prevent the proliferation and survival of cancer cells. Poly(ADP-ribose) polymerase (PARP) inhibitors are a promising novel therapeutic option that prevents cancer progression through this mechanism (10). PARP inhibitors operate by inhibiting the enzymes PARP1 and PARP2, which maintain the integrity of the cellular repair process by facilitating the single-strand break and base excision repair pathways (11). As a result, cells treated with PARP inhibitors develop extensive cellular injury and DNA damage, eventually resulting in cell death. In 2005, a breakthrough study first illustrated the efficacy of single-agent PARP inhibitor treatment with Olaparib in BRCA-mutated ovarian cancer, paving the way for further investigation of PARP inhibitors as a future mainstay of cancer treatment (12). Subsequently, several additional clinical trials expounded on these findings and demonstrated both safety and efficacy of Olaparib in ovarian and breast cancer, with one trial yielding as high as 41% objective response rate in ovarian cancer (13–15). Since these early clinical trials, the efficacy of PARP inhibitors has been even further explored in other cancers, most notably BRCA1/2-deficient cancers due to the increased sensitivity to PARP inhibition that these mutations confer (16, 17) Presently, four PARP inhibitors have been approved by the Food and Drug Administration (FDA) for cancer treatment, specifically ovarian, breast, pancreatic, and prostate cancer: olaparib, niraparib, rucaparib, and talazoparib (**Table 1**) (18, 19).

**Table 1:** Clinically available PARP inhibitors and approved indications.

PARP inhibitor	FDA-approved indications	Key clinical trial evidence	Pharmacologic features	Common adverse effects
Olaparib	Advanced ovarian, breast, pancreatic, and prostate cancer with BRCA mutations or HRD-positive status	Study 19, SOLO-1, POLO, PROfound	Oral; 12-hour half-life; moderate PARP trapping	Fatigue, nausea, anemia, neutropenia
Niraparib	Advanced ovarian cancer (HRD+), HRR-mutated mCRPC (with abiraterone acetate)	QUADRA, MAGNITUDE	Oral; long half-life	Thrombocytopenia, anemia, hypertension
Rucaparib	Recurrent ovarian cancer with BRCA mutations or HRD, mCRPC	Study 10, ARIEL-2, TRITON-2	Oral; primarily renal excretion	Transaminitis, fatigue, anemia
Talazoparib	Germline BRCA-mutated breast cancer; mCRPC (with enzalutamide)	EMBRACA, TALAPRO-2	Oral; highest PARP trapping potency	Anemia, neutropenia, fatigue

Though significant progress has been made in understanding the safety and efficacy of PARP inhibitors in the treatment of the aforementioned cancers with frequent BRCA1/2 mutations, the investigation of cutaneous melanoma and its response to PARP inhibition is poorly understood. Unlike ovarian, breast, pancreatic, and prostate cancer, melanoma has a much lower frequency of BRCA1 and BRCA2 mutations, and there is little evidence of its contribution to melanoma pathogenesis (20). Nonetheless, efforts to investigate the utility of PARP inhibitors in cutaneous melanoma are ongoing. Here, we provide a review of the recent research and clinical trials that have better elucidated the role of PARP inhibitors in melanoma treatment, as well as a discussion of the limitations and considerations that must be taken into account when implementing this treatment for this patient population. We will also suggest avenues for future research to expound on understanding in this field, given the existing literature and current gaps in knowledge on this topic.

## 2 Current use of PARP inhibitors in solid tumors

PARP inhibitors are currently FDA-approved for BRCA1- and BRCA2-mutated breast, ovarian, prostatic, and pancreatic tumors. The synthetic lethality and therapeutic utility of PARP inhibitors in these solid tumors are a result of the relationship between PARP enzymes, base excision repair (BER), BRCA1 and BRCA2 mutations, and HRR pathways. PARP inhibitors primarily target the PARP1 and PARP2 enzymes, which play an important role in resolving single-strand breaks (SSBs) through the BER pathway (11). When DNA sustains SSBs, PARP enzymes are activated and bind to the damaged site (11). Using nicotinamide adenine dinucleotide (NAD+), PARP synthesizes and attaches poly(ADP-ribose) (PAR) chains to itself and other proteins in a process called PARylation (11, 21). This modification recruits DNA repair factors to facilitate the repair of SSBs via the BER pathway. PARP inhibitors block the catalytic domain of PARP enzymes, preventing PARylation and halting the recruitment of repair factors (11). As a result, SSBs accumulate and are converted into double-strand breaks (DSBs) during DNA replication, leading to genomic instability and therapeutic benefit in cancers reliant on defective DNA repair pathways.

Olaparib first became FDA-approved in December 2014 for patients with advanced ovarian cancer positive for BRCA mutations, becoming the first PARP inhibitor to be FDA-approved following the clinical trial Study 19 (NCT00753545) (22). Olaparib significantly improved progression free survival (PFS) compared to the placebo, as the median PFS was 8.4 months compared to 4.8 months in the placebo group. The benefit was most prominent in the subgroup of patients with BRCA mutations, where the median PFS was 11.2 months for Olaparib versus 4.3 months for placebo (14). The SOLO-1 clinical trial results were pivotal in the FDA’s decision to approve Olaparib as first-line maintenance therapy in BRCA mutated advanced ovarian cancer in 2018 (NCT01844986) (23). In May 2020, Olaparib became FDA-approved for first-line maintenance treatment of HRD-positive advanced ovarian cancer in combination with Bevacizumab, a VEGF inhibitor used for anti-angiogenesis (24). Study 10, a Phase II trial, was crucial for the FDA’s approval of Rucaparib, another PARP inhibitor, for advanced ovarian cancer with BRCA mutations. It showed that Rucaparib was adequate, with an overall response rate (ORR) of 53.8% in patients with germline BRCA mutations. It became FDA-approved in patients with BRCA-positive advanced ovarian cancer refractory to two or more prior chemotherapies (25). ARIEL-2, another Phase II trial, demonstrated Rucaparib’s effectiveness in BRCA-mutant and HRD-positive tumors, showing different PFS outcomes based on mutation and HRD status, leading to FDA approval for maintenance therapy in recurrent ovarian cancer (26). In October 2019, Niraparib was approved for use in advanced ovarian, fallopian, and primary peritoneal cancer with HRD-positive status after three or more prior chemotherapy regimens, based on the QUADRA clinical trial results (NCT02354586) (27). Talazoparib was primarily evaluated in breast cancer patients with BRCA mutations. The EMBRACA Phase III trial demonstrated that Talazoparib significantly improved PFS to 8.6 months compared to 5.6 months with standard chemotherapy, leading to its FDA approval in October 2018. Taken together, these results paved the way for the successful clinical use of PARP inhibitors in various solid tumors.

PARP inhibitor use has also been expanded to patients with metastatic castration-resistant prostate cancer (mCRPC) that has been refractory to nonsteroidal antiandrogen medications. The PROfound Phase III clinical trial, NCT02987543, found that Olaparib has reduced the risk of death by 31% in patients with mCRPC (28). Rucaparib has also been FDA-approved for mCRPC after the TRITON-2 clinical trial (NCT02952534). This study supported the approval of Rucaparib for patients with BRCA1/2 or other HRR gene mutations (29). In August 2023, FDA approved AKEEGA (Niraparib and Abiraterone Acetate) for the treatment of patients with BRCA-positive mCRPC based on MAGNITUDE clinical trial (NCT03748641) that has shown improved PFS compared to the placebo group (30). TALAPRO-2, another phase II clinical trial (NCT03395197), compared Talazoparib and enzalutamide combination therapy vs enzalutamide monotherapy in patients, led to FDA-approval of Talazoparib and Enzalutamide combination therapy for patients with or without HRR gene-mutated mCRPC (31). Thus, after extensive investigation into their clinical benefits and potential risks, PARP inhibitors arose as an integral therapeutic option for patients with mCRPC.

Regarding pancreatic cancer, Olaparib is the only PARP inhibitor that has been FDA-approved for usage in patients with pancreatic cancers with BRCA1 or BRCA2 mutations. POLO, a phase III clinical trial (NCT02184195), was crucial for the FDA approval of Olaparib for treating mutant BRCA pancreatic cancer. The trial studied Olaparib in patients with BRCA mutations who were diagnosed with metastatic pancreatic adenocarcinoma and had not progressed after at least 16 weeks of first-line platinum-based chemotherapy and have shown prolonged PFS (7.4 months) compared to the placebo group (3.8months) (32). Other PARP inhibitors, like Niraparib and Rucaparib, have also been studied in pancreatic cancers and have shown clinical benefits and improved median PFS (33–35). In summary, extensive clinical trials and empirical research studies have demonstrated the efficacy of PARP inhibitors in treating breast, ovarian, prostate, and pancreatic cancer, ushering in the acceptance of this treatment regimen as a viable option for targeted solid tumor therapy.

## 3 Use of PARP inhibitors in melanoma

The utility of PARP inhibitors in melanoma has been insufficiently investigated compared to the aforementioned cancers, and novel research is underway to better characterize its efficacy, mechanism of action, and potential limitations. Critical to the advancement of understanding PARP inhibitor use in melanoma was the establishment of HRD prevalence in melanoma. Various metrics for quantifying HRD in cancers exist, ranging from genomic scars, RAD51 foci formation, functional assays, and BRCA1/2 mutation analysis (11). Investigating these biomarkers and more, various studies have been conducted to determine the prevalence of HRD in melanoma as an indicator of responsiveness to various immune checkpoint inhibitors. The frequency of mutations in the HRR pathway in melanoma has been estimated to range from 18% to 57%, with some of the most common mutations involving BRCA1/2, ARM, ARID1A, and BARD1 (36–39). The presence of such mutations is significant, as tumors with HRD are more likely to respond to therapies that exploit DNA repair deficiencies, such as PARP inhibitors. However, the clinical significance of these mutations remains a topic of ongoing investigation, as it is unclear how many of these mutations gives rise to HRD (11). Indeed, there are a wide range of genes implicated in HRR in melanoma, and although mutations in some genes are known to yield favorable responses to targeted cancer therapies, the significance of other mutations remains unclear. Nonetheless, several studies have posited that PARP inhibitors may have utility in melanoma, given the careful determination of patient HRD status prior to treatment (36–38).

On the basis of these findings, several studies have been pursued in order to determine whether HRD status in melanoma patients correlated with ***in vivo*** response to PARP inhibitors as anticipated. A case series by Zhou et al. included both a report of the relationships between response to PARP inhibitors and HRD status of four patients with metastatic melanoma, as well as an analysis of the prevalence of HRD in a cohort of 933 melanoma patients using both genome-wide-LOH (GW-LOH) and traditional direct gene testing and biomarker methods (40). The four patients were all found to have elevated HRD-LOH scores of 43.9%, 57.7%, 32.9%, and 28%, respectively. Each patient was treated with Olaparib at the standard dose twice daily, with patient 3 receiving combination nivolumab and Olaparib. All four patients were found to have positive responses to treatment and no reported adverse events. Regarding the cohort of 933 patients, each modality for determining HRD status yielded a different prevalence of mutation status: GW-LOH with a threshold of 33% found 9% of patients with melanoma were HRD-high, GW-LOH with a 25% threshold found 28%, and using at least one somatic mutation in a DDR gene as a biomarker found 14.7%. Thus, HRD was elevated in all three modes of analysis, corroborating both previous understanding of HRD rates in advanced melanoma and its predicted impact on positive response to PARP inhibition. However, these findings call attention to the variability between HRD-status-measuring modalities, as well as the lack of consensus surrounding appropriate cutoffs for qualifying HRD status. The authors posit that the relatively low HRD scores found in the larger cohort may be a result of the conservative cutoff values used, and emphasize the need to determine a standard LOH threshold for melanoma. In any case, it is imperative that more robust studies with larger sample sizes are conducted to corroborate and offer further insight into these findings, and further explore how they may be leveraged to improve outcomes for melanoma patients.

In addition to using PARP inhibitors as single-agent therapy for melanoma, some studies have expounded on the potential for employing PARP inhibitors as a complement to immune checkpoint blockade (ICB) therapy (**Table 2**). One case study reported the treatment of refractory metastatic melanoma in a 64-year-old male with combination nivolumab and Olaparib (41). Genomic analysis of the lesion found significant HRD and DDR mutations: BRCA2 variant allele frequency (VAF) 24.2%, ATRX VAF 53.5%, TP53 VAF 25.9%, NF1 VAF 25.7%, and GW-LOH 28.4%. Two months after initiation of treatment, the patient demonstrated a complete radiological response of a metastatic liver lesion, clearance of all mutations, and no progression or side effects at the time of publication. Similarly, another case study treated immunotherapy-relapsed cutaneous melanoma in a 42-year-old male with combination nivolumab and Olaparib (42). Genomic analysis of his tumor demonstrated mutations in numerous markers, nominally TERT VAF 73.1%, PIK3CA VAF 24.1%, NRAS VAF 55.5%, and GW-LOH of 32.9%. After two months, the patient demonstrated regression of all lesions — with complete or near-complete resolution of multiple — and mutational clearance. Finally, another case series demonstrated a synergistic therapeutic effect of both MAPK inhibitors and PARP inhibitor combination therapy in three patients with advanced refractory melanoma, who had each failed previous immunotherapy (43).

**Table 2:** Potential strategies for PARP inhibitors in melanoma.

Therapeutic strategy	Mechanistic rationale	Current evidence in melanoma	Limitations and considerations
Monotherapy	Exploits HRD to induce synthetic lethality	Case series showing positive responses in HRD-high metastatic melanoma patients treated with Olaparib	HRD prevalence and thresholds vary; limited large-scale data
Combination with ICBs	ICBs may prevent inhibitory signaling between PD-L1 and PD-1 induced by pro-inflammatory activity of PARP inhibitors	Case reports of nivolumab + Olaparib in immunotherapy-relapsed metastatic melanoma showing complete/near-complete response	Small sample size; exact mechanism driving synergy remains unclear
Combination with MAPK Inhibitors	May have synergistic effect in MAPK inhibitor-resistant melanoma by preventing adaptive resistance	Case series in advanced refractory melanoma reported therapeutic benefit of MAPK + PARP inhibitors	Limited to a few patients; overlapping toxicities need better characterization
Potential Biomarker-Guided Selection Beyond BRCA1/2	Identifies non-BRCA alterations that may predict response (ex: EMSY amplification)	Case report of EMSY-amplified acral melanoma showing near-complete response to talazoparib	Unclear predictive value of rare mutations; needs further validation

**Figure 1:**
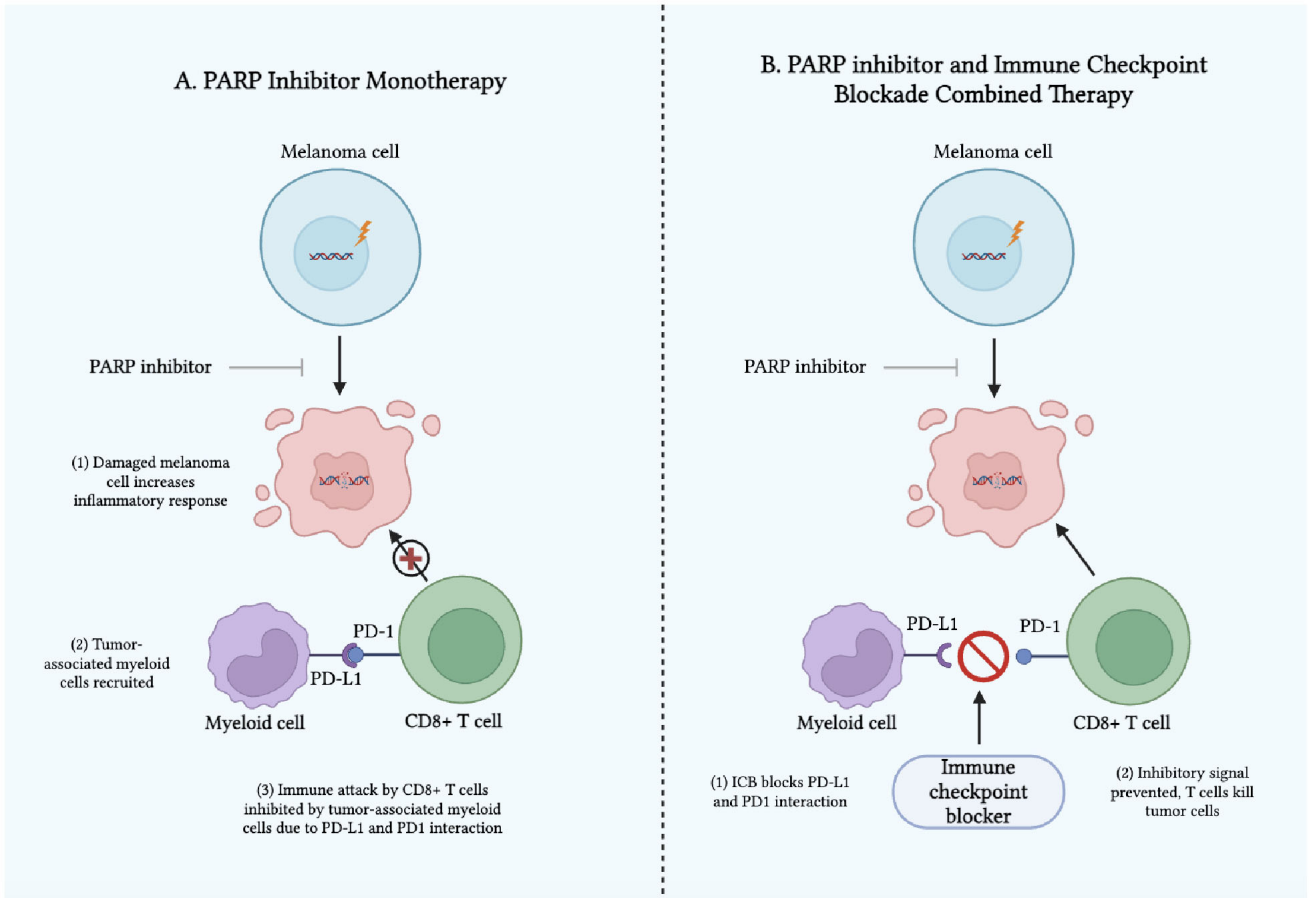
Mechanism of Action of **(A)** PARP Inhibitors as Monotherapy and **(B)** PARP Inhibitors and Immune Checkpoint Blockades as Combined Therapy in Melanoma.

Despite their low statistical power due to limited sample size, the above case studies suggest that PARP inhibitors and ICB used in tandem may offer some potential as a treatment option for advanced or refractory melanoma, though further evidence is needed. The synergistic mechanism between using PARP inhibitors alongside ICB remains poorly understood. One suspected mechanism is that PARP inhibitors potentiate the effects of ICB therapy by amplifying genetic and microenvironment abnormalities, thus making the tumor a more conspicuous target for targeted cancer therapy (41, 42). Another proposed mechanism is that the addition of PARP inhibitors to an ICB regimen exerts its effects via the relationship between PD-L1 expression of myeloid cells and pro-inflammatory activity of PARP inhibitors. While PARP inhibitors generate pro-inflammatory signals that can stimulate anti-tumor immune responses, they also promote the recruitment of myeloid cells (44). These myeloid cells suppress immune activity and contribute to tumor progression by driving the activation of the PD-1/PD-L1 immune checkpoint (44). This dual effect limits the efficacy of PARP inhibitors when used alone (45). However, combining PARP inhibitors with immune checkpoint inhibitors (ICIs) may mitigate this challenge by targeting immunosuppressive myeloid cells and enhancing the overall anti-tumor response, potentially overcoming resistance mechanisms (**Figure 1**) (46). Therefore, this combination therapy approach may harness the immune system to fight the tumor by weakening the genetic profile of melanoma, potentially enhancing patient outcomes (47).

Khaddour et al. also hypothesize that successful treatment with combination PARP inhibitors and ICB may be driven by the PARP inhibitors themselves, given their suggested efficacy against HRD tumors (41). However, the patient in the aforementioned case study notably did not have any mutations in the DDR pathway genes used to determine HRD status, yet still responded positively with combination PARP inhibitor and ICB therapy (42). Thus, the mechanism by which this combination therapy enacts efficacy remains unclear, and these findings underscore the need for a more standardized LOH threshold for melanoma and clear screening criteria for determining potential PARP inhibitor responsiveness. Taken together, these case reports provide encouraging accounts of PARP inhibitors and ICB in advanced melanoma, yet many questions remain about the mechanism driving its propitious effects, and larger-scale studies are essential to truly corroborate such findings.

Beyond HRD and GW-LOH, novel biomarkers to determine PARP inhibitor responsiveness have recently emerged and sparked great research interest (**Table 2**). In another case study, an 88-year-old man with refractory acral lentiginous melanoma and EMSY gene amplification but no abnormal BRCA2 expression was treated with Talazoparib (48). The patient also expressed significant somatic mutations in CCND1, PAK1, and RSF1, as well as GW-LOH of 20.9%, markedly below the standard 33% cutoff. Despite not expressing mutations in the HR-DDR pathways commonly used as indicators of PARP inhibitor responsiveness, this patient demonstrated near-complete responsiveness after 13 months of Talazoparib and expressed a maintained response at 14 months. These findings complement the above discussion of the importance of identifying additional biomarkers and screening criteria to identify patients who may benefit from PARP inhibitors, but may not express the typical mutation profile seen in HRD. As was previously mentioned, a wide range of mutations in both HRR genes and other components implicated in genetic repair exist in melanoma; however, many of these mutations remain of unknown significance given the high mutational burden of melanoma, as well as the large size of several of these implicated genes (45). While it is unclear whether EMSY or the other oncogene mutations were responsible for this patient**’**s response to therapy, these findings speak to the broader need to identify more potential biomarkers for melanoma response to treatment, specifically acral lentiginous melanoma given its lower responses to standard care (49, 50). In all cases, larger, more formal prospective clinical trials are needed to corroborate these findings from case studies with smaller sample sizes, and more systematic demonstration of the utility, mechanism, and safety of these various treatment regimens is critical.

## 4 Key clinical trials of PARP inhibitors in melanoma

A number of notable clinical trials are underway to better characterize the utility of PARP inhibitors in melanoma treatment and identify beneficial therapeutic regimens utilizing PARP inhibitors as either monotherapy or combination therapy. Completed clinical trials of PARP inhibitors in advanced solid tumors have demonstrated variable degrees of effectiveness and safety profiles. Here, we discuss the completed clinical trials examining PARP inhibitors as a treatment modality for melanoma.

A phase II trial (NCT03207347) of Niraparib in patients with BAP1 and other DDR pathway-deficient neoplasms involved 37 patients with advanced tumors (**Table 3**). The study distributed participants into two cohorts: Cohort A (tumors likely to harbor BAP1 mutations) and Cohort B (tumors with other DDR mutations). In Cohort A, the overall response rate (ORR) was 6% partial response (PR) and 44% stable disease (SD), while Cohort B showed an ORR of 0% PR and 43% SD. Common grade 3/4 adverse events (AEs) included anemia (16%), thrombocytopenia (16%), nausea (11%), and vomiting (8%). In spite of the limited ORR, clinical benefit was observed in 78% of patients in Cohort A with confirmed BAP1 mutations, indicating potential for more progress in this subgroup (51). However, the small sample size of patients in this trial with melanoma limits the generalizability of these findings and complicates our ability to extrapolate whether Niraparib would yield clinical benefits for a broader population of patients with melanoma. Furthermore, it is critical to note that BAP1 mutations are more frequent in uveal melanoma than cutaneous melanoma, though they can be found in a subset of cutaneous melanoma (52). The role of BAP1 in cutaneous melanoma is still being studied, and the implications of mutations in this gene on tumor response to targeted cancer therapies remain unclear. Thus, while the findings of this trial are mildly encouraging for the use of Niraparib in BAP1 mutant patients, it is challenging to ascertain its specific utility for cutaneous melanoma. It is imperative that future clinical trials further investigate its efficacy and utility in patients with cutaneous melanoma.

**Table 3:** Clinical trials investigating PARP inhibitors in melanoma.

NCT #	Trial stage	Therapeutic approach	N	Disease/Stage
(NCT00516802)	Phase I (Completed)	Olaparib + Dacarbazine	40	Advanced solid tumors (including stage III or IV melanoma)
(NCT00526617)	Phase I (Completed)	Veliparib + Temozolomide	41	Advanced solid tumors including advanced melanoma
(NCT01618136)	Phase I/II (Completed)	Stenoparib alone or in combination with temozolomide or with carboplatin and paclitaxel	41	Advanced solid tumors or B-cell malignancies
(NCT03207347)	Phase II (Completed)	Niraparib	37	DNA Damage Response Deficient Neoplasms including uveal melanoma
(NCT03925350)	Phase II (Status Unknown)	Niraparib	41	Patients with advanced melanoma with genetic homologous recombination mutations.
(NCT05983237)	Phase I/II (Not Yet Recruiting)	Fluzoparib + Camrelizumab + Temozolomide	50	Unresectable or metastatic stage III or IV melanoma
(NCT01605162)	Phase II (Terminated)	E7016 in Combination With Temozolomide	8	Wild type BRAF stage IV or unresectable stage III melanoma
(NCT04633902)	Phase II (Recruiting)	Olaparib + Pembrolizumab	41	Unresectable or metastatic stage III or IV melanoma with homologous recombination mutations

In another clinical trial, the pharmacokinetics and safety of Veliparib, a potent PARP inhibitor, were assessed in combination with temozolomide in a phase I, open-label, dose-escalation study (NCT00526617) involving 42 patients with nonhematologic malignancies (**Table 3**). Veliparib displayed linear pharmacokinetics and was primarily removed by renal excretion, with no pharmacokinetic interaction observed with temozolomide. Veliparib was well tolerated, and the study authors recommended further investigation in combination therapies to potentially optimize beneficial results in patients with advanced malignancies (53). However, as this trial included a variety of solid tumors, it poses challenges in determining whether the results can be specifically applied to melanoma, given the different underlying mechanisms and responses among cancer types. Furthermore, although this trial offers valuable insight into the pharmacological properties of Veliparib, the clinical response and benefit of this treatment regimen remains unclear with the data provided. Thus, it is critical that future phase II and III clinical trials build off of these findings and address these challenges by more specifically investigating the response of melanoma to this treatment regimen. The potential for PARP inhibitors like Veliparib in melanoma treatment, especially when combined with other therapies, could offer a promising avenue for overcoming resistance mechanisms, but specific clinical trials focused on melanoma will be essential to fully understand its efficacy. By targeting DNA repair pathways in melanoma, which is often resistant to standard therapies, PARP inhibitors could represent a novel therapeutic strategy, but further research is needed to optimize dosing, combination regimens, and patient selection.

In an additional clinical trial of PARP inhibitors in melanoma, a phase I study (NCT00516802) of Olaparib combined with dacarbazine in patients with advanced solid tumors aimed to determine the optimal combination dose for phase II trials (**Table 3**). Out of 40 total patients, the study found that two treated patients (5%) had partial responses, eight patients (20%) had stable disease, and 30 patients progressed (75%) with treatment. Furthermore, both patients who demonstrated partial responses had been previously treated for their melanoma, modestly suggesting the utility of PARP inhibitors in refractory melanoma. Median time to progression was found to be 42 days (95% CI: 36–84 days) for chemotherapy-naïve melanoma patients. The study demonstrated that the optimal dose was 100 mg twice daily Olaparib with 600 mg/m² dacarbazine. Dose-limiting toxicities included neutropenia and thrombocytopenia, with two partial responses observed in patients with melanoma. Although the combination was tolerable, it did not show a clinical advantage over single-agent dacarbazine at these doses. Thus, further inquiry into the toxicities, limitations, and clinical utility of this combination therapy is critical to advancing the use of PARP inhibitors in melanoma (54). Importantly, the inclusion of diverse tumor types again dilutes the specificity of the findings for melanoma patients, and this limitation must be taken into account when interpreting these trial results and considering potential treatment regimens for patients. Additionally, given the concomitant use of dacarbazine alongside Olaparib in this trial, better characterization of the relative contribution of each of these drugs to the therapeutic benefit – as well as the AEs reported – of this therapy regimen is critical. In summary, while the findings of this trial do not strongly suggest a clinical benefit of PARP inhibitors in cutaneous melanoma, they provide valuable insights that can guide future research aimed at optimizing outcomes for this patient population. 

Though limited in number, the completed clinical trials of PARP inhibitors in advanced solid tumors and melanoma are providing valuable insights into the evolving landscape of targeted cancer therapies, which will inform both the personalization and expansion of treatment strategies in melanoma. Niraparib, for example, demonstrated significant benefits in patients with BAP1 mutations, highlighting the critical role of genomic profiling in tailoring therapies. However, it is again critical to note that BAP1 mutations are more prevalent and have greater prognostic relevance in uveal melanoma compared to cutaneous melanoma, suggesting that the therapeutic potential of Niraparib in cutaneous melanoma may be more limited. Similarly, Veliparib showed promising antitumor activity and tolerability, paving the way for potential combination strategies, which could enhance its efficacy in melanoma treatment. However, the generalizability of these findings to melanoma patients requires further validation through more focused clinical trials examining how Veliparib interacts with melanoma**’**s unique molecular characteristics. Lastly, while the combination of Olaparib and dacarbazine needs modification, it exemplifies the growing interest in innovative drug pairings, underscoring the need for more strategic combinations to overcome melanoma**’**s resistance mechanisms. Ongoing and recruiting clinical trials are increasingly refining patient selection, optimizing dosages, and addressing resistance pathways, which could ultimately unlock the full potential of PARP inhibitors in melanoma. A summary of the key clinical trials – both completed and active – investigating the use of PARP inhibitors in melanoma is presented below (**Table 3**).

## 5 Discussion

Despite several advancements in the field of cancer therapeutics, melanoma remains an incredibly challenging malignancy to treat, particularly in its advanced stages. Not only do many patients with melanoma experience disease progression and relapse despite the development of more sophisticated therapeutic options, but many develop resistance to treatment as well (55). Thus, the advent of PARP inhibitors in the field of melanoma treatment has brought much excitement and potential to the field of targeted cancer therapy, particularly for patients with clinically significant genetic alterations, such as HRD and BRCA1/2 mutations. By selectively targeting cancer cells and their DDR pathways, PARP inhibitors increase tumor mutation burden and neoantigen production, causing the tumor to be more susceptible to immunotherapy response (11). Thus, PARP inhibitors may offer a novel, personalized approach to treating patients with advanced or refractory melanoma. Despite the promising potential of PARP inhibitors in melanoma treatment, the majority of studies conducted thus far have been case reports or small clinical trials, often with limited sample sizes. To establish a solid foundation for their widespread use, larger, well-designed clinical trials are needed to better understand their efficacy and safety in broader melanoma populations.

Though progress is steadily being made in elucidating the efficacy of PARP inhibitors in melanoma, several key challenges and questions regarding their clinical utility remain (**Table 4**). Namely, there is a need to identify novel biomarkers that can predict patient response to PARP inhibitors. While the efficacy of PARP inhibitors was first demonstrated against solid tumors with BRCA1/2 mutations and HRR defects, these alterations are not universally present in melanoma patients given the molecular heterogeneity of melanoma (56). Thus, there is a need to identify additional predictive biomarkers for PARP inhibitor response. Some potential targets currently under investigation include other components of HRR and DNA repair, including ATM, ATR, and CHK1. Indeed, recent studies have shown that patients with melanoma who develop resistance to MAPK inhibitors display notable sensitivity to PARP inhibitors (57, 58). The proposed mechanism is due to the downregulation of ATM genes, which repair double-stranded DNA breaks and activate DNA repair genes under normal conditions (57, 59). As ATM becomes downregulated, this decreases overall tumor cells’ ability to detect damaged DNA, likely making them more susceptible to PARP inhibitors (57). This suggests that a deeper understanding of the underlying mechanisms of resistance and the role of DNA repair pathways could enhance the personalized application of PARP inhibitors, particularly in cutaneous melanoma where treatment options are limited and resistance to standard therapies is a barrier. These findings highlight the importance of identifying and continuing to characterize novel biomarkers in future studies, which may improve the clinical utility of PARP inhibitors in melanoma therapy.

**Table 4:** Challenges in Implementing PARP Inhibitors as Melanoma Treatment.

Challenge	Underlying Cause	Clinical Implication	Potential Mitigation Strategy
Low prevalence and heterogeneity of HRD in melanoma	Melanoma has fewer BRCA1/2 mutations compared to ovarian/breast cancers; HRD cutoff thresholds remain poorly standardized	Difficulty identifying patients most likely to respond; risk of overtreatment in HRD-low tumors	Development of validated melanoma-specific HRD biomarkers and standardized genomic assays
Unclear optimal patient selection	Lack of consensus on genomic predictors beyond BRCA1/2 (ex: EMSY)	Uncertain efficacy in HRD-intermediate melanoma	Expand biomarker discovery and functional HRD assays
Adverse events and tolerability	Wide variety of AEs, including hematologic toxicity, gastrointestinal effects, rare cardiovascular risks	Limits feasibility as long-term therapy; commonly a patient concern	Optimized dosing schedules; supportive care strategies
Drug resistance development	BRCA reversion mutations, replication fork stabilization, drug efflux, alternative DDR pathway activation (ATR/CHK1)	Limits durability of response	Combination therapies (ATR or WEE1 inhibitors) and sequential treatment approaches
High cost and access barriers	Monthly PARP inhibitor cost is roughly $13,000-15,000; limited insurance coverage	Reduced patient access; higher risk of non-adherence	Financial assistance programs; policy changes to improve affordability
Limited melanoma-specific clinical trial data	Existing evidence largely from case series and small cohorts	Uncertain efficacy compared to other therapies	Larger, melanoma-specific focused phase II/III trials with HRD stratification

Another critical avenue for advancing the use of PARP inhibitors in melanoma is the continued investigation of their combination with ICB therapy. As mentioned previously, the precise mechanism of the synergism of PARP inhibitors and ICB combined therapy is not fully understood, but several potential molecular bases behind this treatment approach have been proposed. Accurately characterizing the mechanism of combined PARP inhibitor and ICB therapy will enable the accurate development and implementation of targeted therapies and likely yield more beneficial clinical outcomes for patients. Specifically, continued exploration of the optimal sequencing, dosing, patient selection, and potential toxicities of PARP inhibitor and ICB combinations is critical. Of note, a recruiting phase II clinical trial (NCT04633902) will evaluate the use of pembrolizumab with Olaparib and its clinical efficacy in treating metastatic melanoma that has been refractory to ICI and BRAF inhibitors treatment (60). Another phase I/II clinical trial (NCT05983237), which is not yet recruiting, will investigate Fluzoparib in combination with Camrelizumab and temozolomide in advanced melanoma (61). The results of these trials, along with others exploring the integration of PARP inhibitors and ICB, could lead to improved outcomes for many melanoma patients, particularly those with refractory or advanced disease.

As we continue to discover more about PARP inhibitors and expand their utilization to more patients with melanoma, several considerations and limitations of this treatment must be kept in mind. Although PARP inhibitors are generally well-tolerated, numerous clinical trials have reported AEs, including anemia, fatigue, gastrointestinal symptoms, and hematological adverse events, likely due to the direct target of PARP1 and PARP2 (62). Talazoparib has been associated with hematopoietic AEs, leading to dose modification in Phase II (ABRAZO) and Phase III (EMBRACA) trials (63). Additionally, increased risk of cardiovascular and thrombolytic events has been reported in patients who receive PARP inhibitors, namely Niraparib (64). It is imperative that future clinical trials seek to minimize or eliminate such AEs to ensure patient safety and satisfaction. Given these AEs, cutaneous melanoma patients may choose to opt for other therapeutic avenues, and it is critical that providers are transparent about the risks and novel status of PARP inhibitors in the current treatment landscape.

Another important challenge to address is resistance to PARP inhibitors. It has been estimated that 40–70% of patients with other solid tumors are likely to develop resistance while using PARP inhibitors as monotherapy for cancer treatment (65). Multiple mechanisms of resistance have been proposed, including genomic reversal of BRCA1 and BRCA2 which restores HRR, epigenetic modifications, restoration of replication fork protection via FANCD2 and RAD51, and upregulation of drug efflux transporters such as ABCB1P-glycoprotein (66). These adaptations, either singularly or in combination, may allow melanoma cells to bypass the synthetic lethatlity induced by PARP inhibitors and regain genomic stability, thus rendering treatment ineffective. Understanding the molecular underpinnings of these resistance mechanisms is essential for developing strategies to overcome resistance, ensuring durable responses, and improving patient outcomes. Tackling these resistance mechanisms may require combination approaches, such as pairing PARP inhibitors with ATR or WEE1 inhibitors to prevent replication stress recovery, or with checkpoint inhibitors to enhance tumor immunogenicity (57, 58).

Finally, the social determinants impacting the accessibility and utilization of PARP inhibitors are critical to consider and discuss. PARP inhibitors, while promising from a therapeutic standpoint, are often very expensive, and can impose a significant financial burden on patients seeking treatment. Monthly total drug costs for PARP inhibitors reportedly range from $13,000 to $15,000, and cost-effectiveness studies have found that cost-effectiveness ratios for PARP inhibitors typically fall above accepted willingness to pay thresholds, further demonstrating their high cost (67). However, the utilization of financial assistance programs can help mitigate some of this cost, as another study investigating PARP inhibitor cost in a population of 76 patients found that the average monthly out of pocket (OOP) cost for patients was $46, with financial assistance programs contributing an average of $358 per month and payors contributing an average of $12,019 per month (68). Thus, although the cost of PARP inhibitors remains high, financial assistance programs have the potential to improve accessibility of these drugs for patients in need. Such social determinants are important to address, as evidence has emerged that higher patient OOP costs are associated with higher rates of prescription abandonment, delayed initiation of treatment, and non-adherence, which can all compound and lead to worsened patient outcomes (67). Thus, future research initiative should not only address the clinical efficacy of PARP inhibitors, but also on means to reduce their cost, improve accessibility, and mitigate the social determinants of health that patients may face when receiving treatment.

## 6 Conclusion

The use of PARP inhibitors for the treatment of melanoma is an actively-evolving area of investigation and discovery. Though these agents have long demonstrated efficacy in the management of ovarian, breast, prostate, and pancreatic cancers, their newly-established utility in the treatment of melanoma has brought promise to the field of cancer therapeutics. Although early studies and clinical trials have yielded modestly encouraging results, several challenges remain before PARP inhibitors can become mainstays of cancer therapy. Mechanisms of resistance, novel biomarkers for patient selection, and beneficial combination therapies with PARP inhibitors and ICB therapy are among the foremost areas of research that must be further explored and characterized to optimize the use of PARP inhibitors in melanoma and limit AEs. Additionally, the high cost of PARP inhibitors, coupled with the social determinants of health that patients face, underscore the need for policy initiatives and financial assistance programs. Continued research is essential to better understand the most effective ways to integrate PARP inhibitors into existing treatment paradigms, identify patients most likely to benefit, and address challenges such as drug resistance. As our understanding of melanoma’s molecular landscape evolves, PARP inhibitors may become a valuable component of the management of these tumors. As of now, however, PARP inhibitors remain largely experimental in the context of melanoma, and their routine clinical use is limited by unresolved questions regarding optimal patient selection, resistance mechanisms, and long-term efficacy.
